# Biomarkers for Premature Coronary Artery Disease (PCAD): A Case Control Study

**DOI:** 10.3390/diagnostics13020188

**Published:** 2023-01-04

**Authors:** Muhammad Faizan A. Shukor, Qurratu Aini Musthafa, Yasmin Anum Mohd Yusof, Wan Zurinah Wan Ngah, Noor Akmal Shareela Ismail

**Affiliations:** 1 Chemical Defense Research Center, National Defense University of Malaysia, Kuala Lumpur 57000, Malaysia; 2 Department of Biochemistry, Faculty of Medicine, Universiti Kebangsaan Malaysia (UKM), Kuala Lumpur 56000, Malaysia; 3 Department of Biochemistry, Faculty of Medicine, National Defense University of Malaysia, Kuala Lumpur 57000, Malaysia

**Keywords:** biomarkers, premature coronary artery disease, atherosclerosis, heart disease, risk prediction

## Abstract

Coronary artery disease (CAD) is often associated with the older generation. However, in recent years, there is an increasing trend in the prevalence of CAD among the younger population; this is known as premature CAD. Although biomarkers for CAD have been established, there are limited studies focusing on premature CAD especially among the Malay male population. Thus, the aim of this research was to compare the biomarkers between premature CAD (PCAD) and older CAD (OCAD) among Malay males. Subjects, recruited from the Universiti Kebangsaan Malaysia Medical Centre and National Heart Institution, were divided into four groups: healthy control < 45 years old; premature CAD (PCAD) < 45 years old; healthy control > 60 years old; and older CAD (OCAD) > 60 years old, with n = 30 for each group. Ten potential markers for CAD including soluble sVCAM-1, sICAM-1, interleukin-2, interleukin-6, interleukin-10, Apo-E and Apo-A1, homocysteine, CRP, and vitamin D levels were examined. Our results revealed premature CAD patients had significantly higher values (*p* < 0.05) of sVCAM-1, CRP, interleukin-6, and vitamin D when compared to the age-matched controls. Similarly, older CAD patients showed higher levels of sVCAM-1, CRP, and interleukin-2 when compared to their age-matched controls. After adjusting for multiple parameters, only CRP remained significant for PCAD and interleukin-2 remained significant for CAD. This indicates that premature CAD and older CAD patients showed different profiles of protein biomarkers. CRP has the potential to become a biomarker for premature CAD while interleukin-2 is a better biomarker for older CAD together with other typical panels of protein biomarkers.

## 1. Introduction

Coronary artery disease (CAD) is one of the major causes of death in the world. It is commonly associated with the older population. However, about 3–10% of CAD cases involve persons aged 45 years and below; this cohort has been identified as premature CAD (PCAD) [1]. The numbers appear to be fairly constant each year, suggesting that there could be strong genetic contributions to PCAD development beyond conventional risk factors [2]. Although epidemiological studies have found a low prevalence for PCAD, it is possible that these numbers are underestimated. In fact, 10% of CAD cases already represent more than 700,000 cases around the world (WHO, 2016). Moreover, increasing rates of smoking and obesity among young adults could contribute to the rise in PCAD cases [3]. In Malaysia, a national survey revealed CAD as the leading cause of cardiovascular disease (CVD) [4] that affects older generations [5].

The pathogenesis of CAD has been linked with endothelial dysfunction, dyslipidemia, inflammation, and oxidative stress [6]. During disease progression, proteins such as inflammatory cytokines and apolipoproteins as well as pro- and antioxidant molecules contribute by facilitating atherogenesis. Several studies have extensively elucidated certain biomarkers including sVCAM-1 [7,8,9], sICAM-1 [8,9], IL-2 [10], IL6 [11], apolipoprotein E [12,13], and apolipoprotein A1 [14]. Thus, all of these proteins have the potential to become biomarkers for CAD. A recent guideline for risk assessment of CAD was proposed by The American College of Cardiology and the American Heart Association (ACC/AHA) [15]. This guideline includes an assessment of adults who are 40 to 75 years old by undergoing a 10-year atherosclerotic cardiovascular disease (ASCVD) risk estimation, which includes family history, hsCRP, and coronary artery calcium scores for CAD diagnosis. Various studies have suggested different types of diagnostic marker for CAD such as homocysteine, fibrinogen, lipoprotein phospholipase A2, and interleukin-1 receptor-like 1 [16,17]. However, there is an emerging literature reporting on specific predictors for PCAD cases. Although the mechanisms involved in the pathogenesis of older CAD (OCAD) and PCAD are similar, a study illustrated that there are differences in their plaque morphology and its constituent components [18]. Such findings raise a question of whether the biomarkers for OCAD are also applicable for PCAD. Therefore, there is some urgency to identify specific and sensitive biomarkers that give a better risk assessment to differentiate between PCAD and OCAD cases.

Thus, this study intended to determine the levels of several plasma proteins that are involved in inflammation and lipid metabolism such as soluble vascular cell adhesion molecule 1 (sVCAM-1), soluble intercellular adhesion molecule 1 (sICAM-1), interleukin-2 (IL-2), interleukin-6 (IL-6), interleukin-10 (IL-10), apolipoprotein E (Apo-E), and apolipoprotein A1 (Apo-A1). Additionally, we also included other established CAD biomarkers such as homocysteine, CRP, and vitamin D to further supplement the findings of this study. Thus, this study aimed to identify specific biomarkers that are associated with the PCAD and OCAD populations and identify the discernable differences in both categories.

## 2. Materials and Methods

### 2.1. Study Population

This study was approved by the IJN Scientific and Ethics Committees (IJNEC/01/2012 5) and UKM Research Ethics Committee (UKM 1.5.3.5/244/UMBI-001-2012). All subjects recruited into this study gave their written consent to participate in this study. A total of 120 subjects were divided into four groups, with 30 subjects in each group, i.e., (1) PCAD patients aged below 45 years old (PCAD), (2) CAD patients aged above 60 years old (OCAD), (3) healthy controls aged below 45 years old (C45), and (4) healthy controls aged above 60 years old (C60). The PCAD and OCAD subjects were recruited from patients presenting to the National Heart Institute of Malaysia (IJN) and UKM Medical Centre (UKMMC) while healthy controls subjects were recruited from the Malay community in Selangor and Klang Valley. The number of samples needed was determined using Power and Sample Size Calculation software [19]. Significant values were set at 0.05, and the ratio between case and control was set at 1. Concentrations and standard deviations for parameters including SOD activity and VCAM-1 and homocysteine levels were taken from previous studies [20,21,22]. The minimum sample size required was 10, and 30 subjects per group were shown to be more than enough to obtain a study power of 0.8. The Malay race represents the highest proportion of races in the population in Malaysia; thus, that was the reason for its recruitment in this study. It is also known that the prevalence of Malay males getting CAD due to hypertension and hypertriglyceridemia is higher than that of the other races [4,5,23].

**Figure 1:**
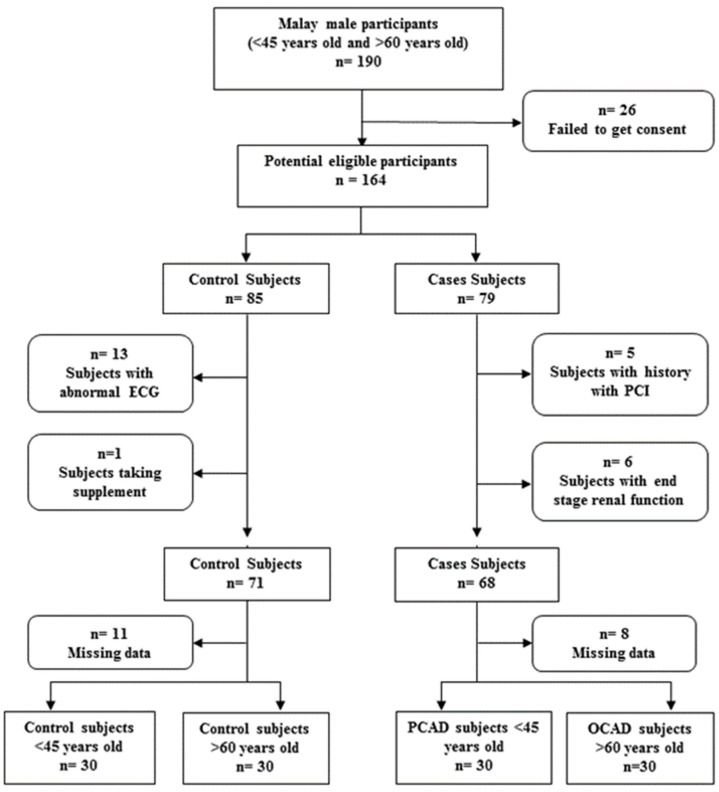
Flow chart for subject selection. Final number of subjects selected was 30 for each group.

Thereby, the PCAD and OCAD subjects selected for this study were Malay males possessing more than 70% stenosis in one or more of their major coronary arteries detected during angiography. The controls selected aged below 45 years old and above 60 years old were selected among healthy Malay males with a normal electrocardiogram (ECG) from Nihon Kohden (Tokyo, Japan) and age matched to the PCAD and OCAD subjects. We excluded subjects aged between 45 and 60 years old to provide extreme differences between younger and older subjects. Those who went for angiograms, although with normal results, were not considered as healthy subjects as they were already symptomatic. Females and non-Malay males were excluded to increase the homogeneity of our subjects. Blood samples were taken prior to angiography or PCI procedure. The control subjects underwent an ECG to exclude any asymptomatic cardiac rhythm anomalies in addition to the assessment of the lack of any personal history indicative of CAD. Age, body mass index (BMI), and smoking habits for all subjects were either obtained from their medical records or during an interview with the subject. Smoking status was defined as current smoker, while an individual who was not smoking or had quit for more than a year was defined as non-smoker. All subjects were required to fast for at least 6 h prior to blood sample collections. The flow chart for subject selection is shown in Figure 1.

### 2.2. Demographic and Biochemical Analysis

ECG was performed by qualified medical doctors from the UKM Medical Centre. Biochemical data for the PCAD and OCAD subjects were collected from their medical records while a routine biochemical analysis for healthy controls was performed by Quantum Diagnostics Sdn Bhd (Selangor, Malaysia). Lipid, renal, and liver profiles were obtained as standard biochemical parameters in this study.

### 2.3. Determination of Heart Biomarkers

Blood samples drawn from subjects (15 mL) were placed into EDTA tubes (BD Vacutainer, Becton, Dickinson and Company, Plymouth, UK). These blood samples were separated into peripheral blood mononuclear cells (PBMC), red blood cells, and plasma through centrifugation at 1800× *g* for 30 min at 4 °C. The components were subsequently stored at −80 °C prior to analysis.

Plasma sVCAM-1, sICAM-1, IL-2, IL-6, and IL-10 levels were analyzed using a Procarta 5 plex kit (Affymetrix, Santa Clara, CA, USA), while plasma Apo-A1 and Apo-E levels were determined by using a Procarta 2 plex kit (Affymetrix, USA). Homocysteine, vitamin D, and CRP concentrations were measured using an ELISA kit from IBL International GmbH (Hamburg, Germany), Immunodiagnostic (Frankfurt, Germany), and Biovendor (Brno, Czech Republic), respectively. All procedures were performed according to the protocols provided by each of the manufacturers. 

### 2.4. Statistical Analysis

Data were analyzed using SPSS software version 16.0 for Windows. Data for continuous variables were presented as mean ± standard deviation, and categorical data were summarized as percentages. Normally distributed data were analyzed using the *t*-test, while the Mann–Whitney test was performed for non-normally distributed data. The Shapiro–Wilk test was used to determine the normality of our data. Extreme values as indicated by SPSS were excluded from the analysis. The results were considered significant if the *p*-value was less than 0.05 (*p* < 0.05). Bivariate and binary logistic regression analyses were also performed. The sVCAM-1, IL-6, vitamin D, CRP, and Apo-A1 were selected as variables in the younger groups, while sVCAM-1, IL-2, Apo-A1, Apo-E, and CRP were selected as variables in the older groups for binary logistic analysis. Variables were selected based on their significant differences between cases and controls as initially determined by a *t*-test or Mann–Whitney test. Statistical analysis was not adjusted for medication to closely represent real situations where individuals presenting for screening are most likely on some sort of medication. Therefore, our proposed biomarkers might be useful in predicting CAD regardless of medications used.

## 3. Results

We first looked at the biochemical parameters of both the PCAD and OCAD subjects while comparing them to the healthy, age-matched controls. In PCAD, a significantly higher level was seen with fasting glucose, triglyceride, potassium, and ALT, while OCAD showed significantly higher fasting glucose, potassium, and urea levels when compared to their age-matched controls. Reduced HDL and sodium levels were observed in the PCAD subjects while the OCAD subjects have significantly lower LDL, total cholesterol, and sodium levels compared to their age-matched controls. The subjects’ demographic and biochemical profiles are shown in Table 1.

**Figure 2:**
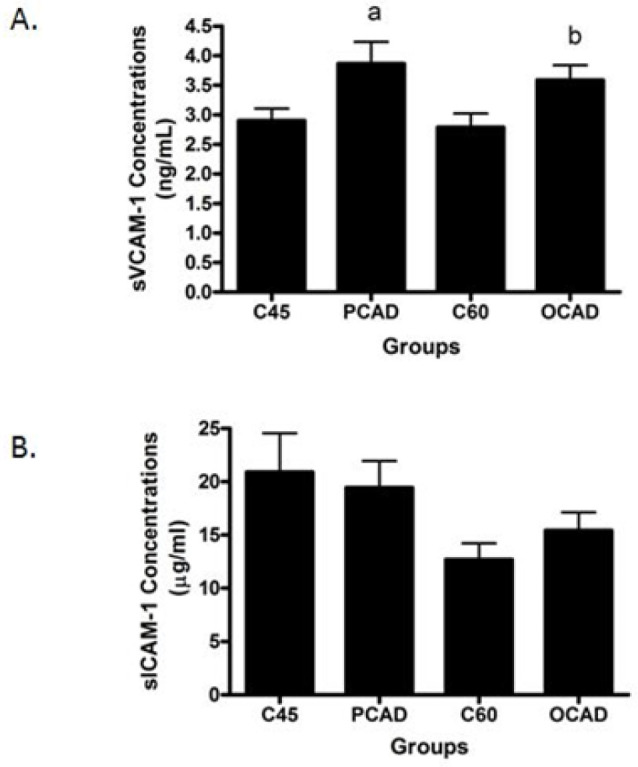
(**A**) The sVCAM-1 concentrations and (**B**) sICAM-1 in cases and control subjects. ^a^ *p* = 0.026 compared to C45 and ^b^ *p* = 0.023 compared to C60. The sVCAM-1 was analyzed using a *t*-test while sICAM-1 was analyzed using a Mann–Whitey test.

**Figure 3:**
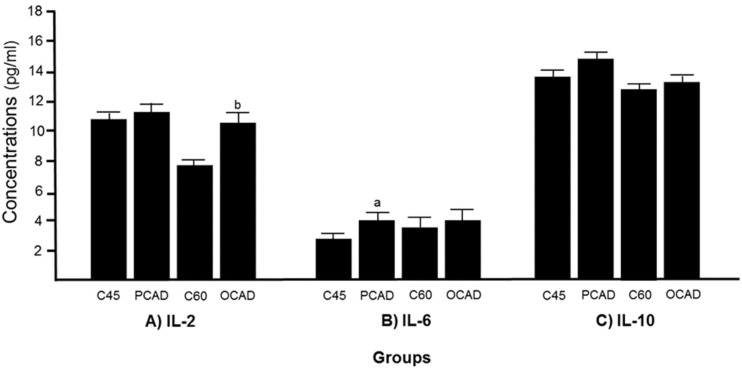
The concentration levels of A) IL-2, B) IL-6, and C) IL-10 in cases and control subjects. ^a^ *p* = 0.016 compared to C45; ^b^ *p* = 0.012 compared to C60. IL-2, IL-6, and IL-10 were analyzed using a *t*-test.

**Figure 4:**
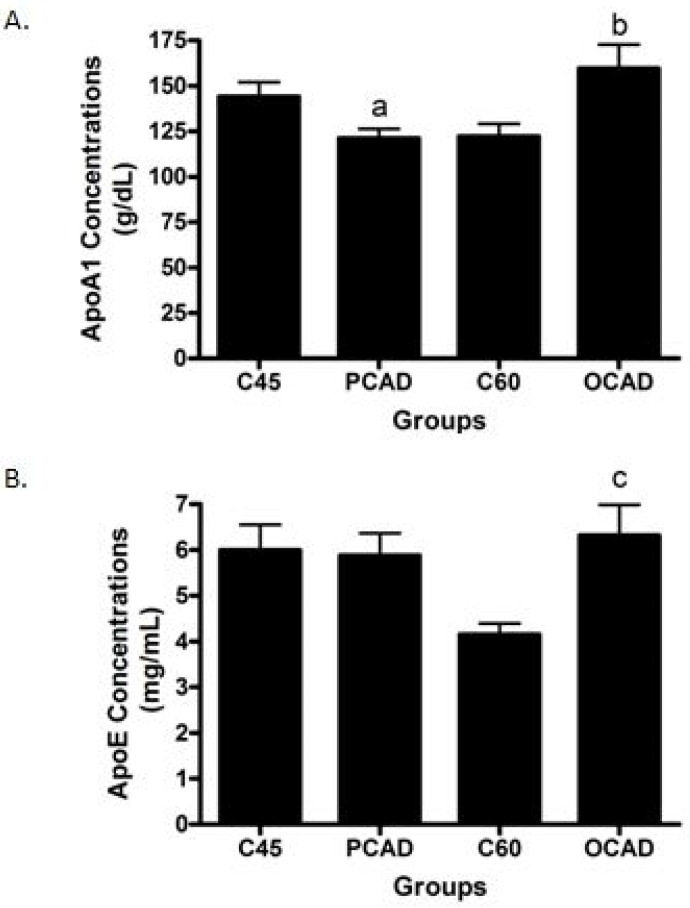
(**A**) Apo-A1 and (**B**) Apo-E concentrations in cases and control subjects. ^a^ *p* = 0.019 compared to C45; ^b^ *p* = 0.016 compared to C60; and ^c^ *p* = 0.004 compared to C60. Apo-A1 and Apo-E were analyzed using a *t*-test.

**Figure 5:**
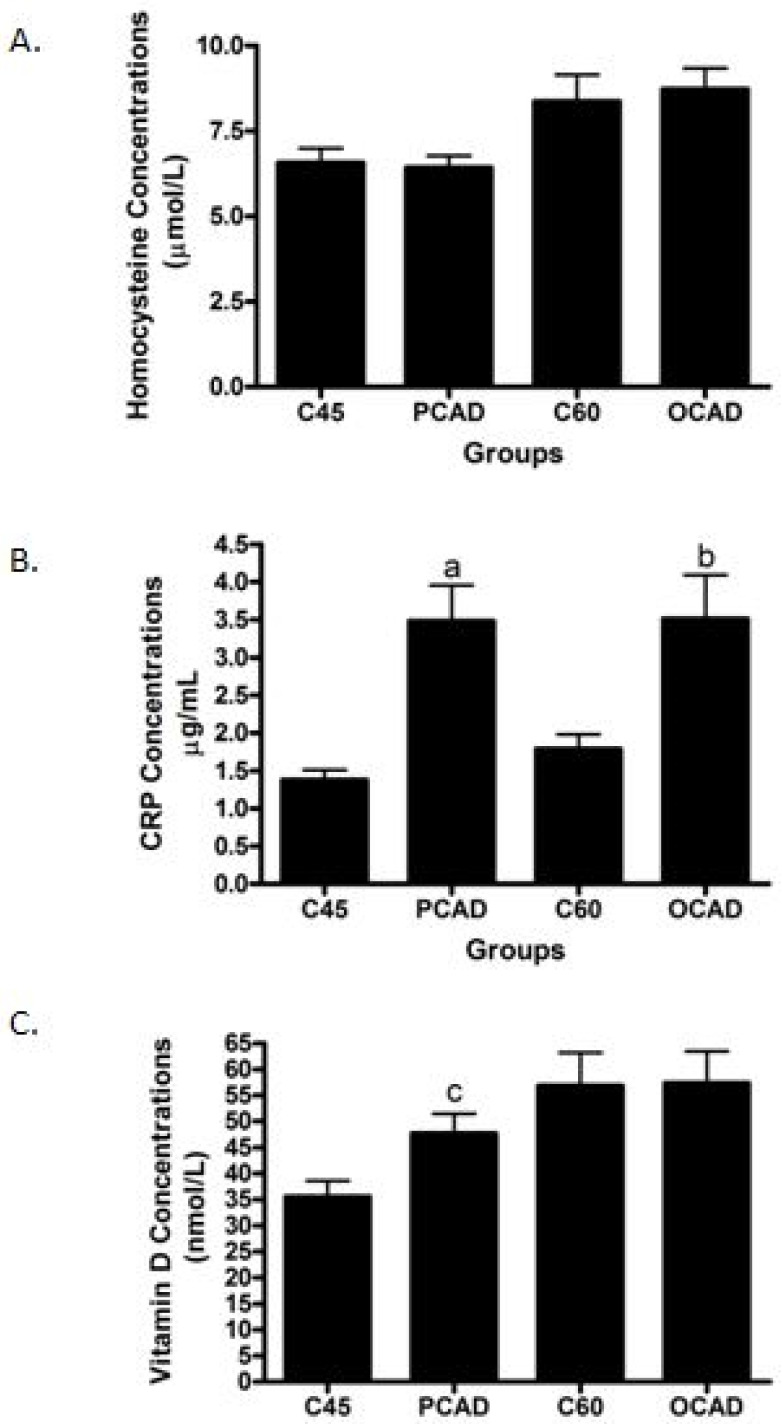
The concentration levels of (**A**) homocysteine, (**B**) CRP, and (**C**) vitamin D in PCAD and OCAD cases and control subjects. ^a^ *p* < 0.001 compared to C45 using *t*-test analysis; ^b^ *p* = 0.016 compared to C60 using Mann–Whitney analysis; and ^c^ *p* = 0.016 using *t*-test analysis.

Next, we scrutinized the potential biomarkers for CAD. The sVCAM-1 concentrations were found to be significantly higher in both the PCAD and OCAD subjects when compared to the age-matched controls (Figure 2A), while sICAM-1 was unchanged (Figure 2B). Further analysis revealed that while the OCAD subjects had significantly higher IL-6 (Figure 3A), the PCAD subjects also had significantly higher IL-6 (Figure 3B) when compared to their age-matched controls. Plasma IL-10 levels showed no significant difference between the groups (Figure 3C). Plasma Apo-A1 concentrations decreased significantly in the PCAD subjects as compared to the age-matched controls (Figure 4A). In contrast, Apo-A1 levels were higher in the OCAD subjects when compared to their age-matched controls (Figure 4A). The same pattern was observed in the OCAD patients for Apo-E levels (Figure 4B). The OCAD patients also exhibited significantly higher levels of homocysteine compared to the PCAD subjects (Figure 5A), but no significant difference was noted when compared to their age-matched groups. Plasma CRP levels were also seen to be significantly higher in both the PCAD and OCAD subjects when compared to the age-matched controls (Figure 5B). Only the PCAD subjects showed significantly higher vitamin D levels when compared to the age-matched controls (Figure 5C).

Subsequently, we conducted a correlation analysis between the biomarkers amongst the PCAD subjects. Plasma sVCAM-1 was found to be positively correlated with IL-6 (r = 0.63) and IL-10 (r = 0.734). Plasma IL-6 also showed a positive association with homocysteine (r = 0.477,) and IL-10 levels (r = 0.436). Furthermore, significant positive associations were found between plasma Apo-A1 with IL-2 (r = 0.472) and Apo-E levels (r = 0.583) and between plasma vitamin D with IL-10 levels (r = 0.436). In the OCAD group, positive correlations were found between plasma homocysteine with Apo-E (r = 0.584) and plasma sVCAM-1 with IL-10 levels (r = 0.562). Correlation analysis for all parameters among the PCAD and OCAD subjects are shown in Table 2 and Table 3, respectively.

We performed a logistic regression to ascertain the effects of plasma levels of sVCAM-1, IL-6, vitamin D, CRP, and Apo-A1 on the likelihood of having PCAD. The logistic regression model was shown to be statistically significant (χ2 = 20.05, df = 5, *p* < 0.001), explained 52% (Nagelkerke R^2^) of the variation, and correctly classified 78% of the cases. Subjects with higher CRP levels were found to be 2.47 times more likely to have PCAD (CI: 1.004–6.055). For the OCAD subjects, adjusting for plasma sVCAM-1, IL-2, Apo-A1, Apo-E, and CRP, the model was found to be statistically significant with χ2 = 27.97, df = 5, and *p* < 0.001, explained 67% (Nagelkerke R^2^) of the variation, and correctly classified 82.5% of the cases. Plasma IL-2 was found to be the only significant variable with OR = 1.64 (CI: 1.06–2.537).

## 4. Discussion

Predicting risk and development of CAD is a challenging process. The use of traditional risk factors such as smoking [24], cholesterol [25], and blood pressure [26] exhibits mixed findings in correlating with CAD among individuals. As CAD is a complex disease involving multiple processes as well as possessing interactions among genes, proteins, and the environment, relying on traditional risk factors alone may not provide an accurate assessment. While earlier extensive studies only focused on the CAD population in general or among the elderly, there is a priority need to study risk assessment in the younger population, which is somewhat neglected at present. However, emerging data have slowly elucidated that several protein biomarkers are able to predict PCAD cases. Thus, to enrich knowledge of the current trend, our study showed and highlighted several potential protein biomarkers useful for predicting PCAD and OCAD and also elucidated their relationships and correlations between these two types of heart diseases.

In this study, subjects were divided into four groups based on their age and disease status. Subjects between 45 and 60 years old were excluded to ensure a clear demarcation between younger and older subjects. Our case subjects were those having coronary artery stenosis confirmed by angiography, while our healthy controls were those asymptomatic for CAD and other diseases and without any cardiac rhythm disorders as determined by ECG. We are fully aware that ECG alone is not able to confirm if an individual suffers from any degree of stenosis of their coronary vessels. Having control subjects undergo an angiogram in order to exclude vessel stenosis when they were without any evidence of CAD was not agreed to by the cardiologists at both the study site hospitals and was deemed unethical. As such, the study design only considered CAD cases with significant stenosis, encompassing 70% of the vessel lumen, as being eligible. Nevertheless, this same method of selecting sample populations was used in another study and the results of that study have already been published [27].

We observed that both the older groups had higher systolic blood pressure compared to the younger groups, which was expected as increasing age is correlated with an increase in blood pressure [28]. The percentage for smoking was lower in the older groups, suggesting there is a shift towards a healthier lifestyle with increasing age. Lipid profile showed that the PCAD group had higher Tg levels but lower HDL when compared to the age-matched controls. However, the OCAD patients had lower LDL and cholesterol levels compared to their age-matched controls. The differences in lipid profile were probably due to the dietary habits and the influence of lipid-lowering drugs. Indeed, statin has been shown to inhibit HMG-CoA reductase, thereby decreasing the cholesterol and LDL levels in blood [29]. Although renal profiles and liver function tests showed significant differences between cases and their age-matched controls in some of the parameters, the values were still within the normal range, suggesting these differences will not affect PCAD or OCAD development.

Our data indicated a significant increase in plasma sVCAM-1 in both the PCAD and OCAD subjects, but not for sICAM-1. Both sVCAM-1 and sICAM-1 are adhesion molecules that mediate monocyte migration into the subendothelial space. The expression of these proteins was induced by inflammatory factors such as TNF-α [30]. Our finding was in line with previous studies, which suggested sVCAM-1 was a better marker than sICAM-1 in CAD cases [7,9,21,31]. Furthermore, we also observed a higher level of IL-6 exclusively in the PCAD subjects, while IL-2 levels were higher in the OCAD subjects, suggesting a different profile of inflammatory markers that may be related to age differences. IL-2 appears to be an independent predictor for OCAD even after adjusting for multiple variables. IL-2, -6, and -10 are known to be actively involved in inflammatory responses. For instance, IL-2 is highly expressed in atherosclerotic plaques, and its concentration has been found to be increased in CAD cases [10,32] especially in unstable angina subjects [33]. It is believed that IL-2 is a pro-atherogenic cytokine due to its ability to promote the differentiation of Th cells towards Th1 phenotypes [10,34]. Higher concentrations of IL-6 also have been observed in atherosclerosis patients [35]. It functions primarily in inducing acute phase reaction, activation of endothelium, and promoting lymphocyte differentiation [36]. Conversely, IL-10 is an anti-atherogenic cytokine. In atherogenesis, IL-10 inhibits macrophage activation and secretion of inflammatory cytokines such as IL-1B, TNF-α, and IL-8 [37] through the inhibition of NF-κB activity [38]. However, we did not find any significant changes in IL-10 among both the PCAD and OCAD subjects, which coincides with a Mexican study that found the IL-10 allele (rs1800896) is associated with a decreased risk of developing PCAD [39].

Interestingly, we found a positive correlation between sVCAM-1 and IL-10 in both PCAD and CAD cases. Fiehn and colleagues demonstrated that IL-10 treatment on endothelial cells did not have any effect on sVCAM-1 expressions [40]. However, after co-cultivation with activated leukocytes, sVCAM-1 expression was increased after treatment with IL-10 [40]. The possible mechanism involved is that IL-10 increases sVCAM-1 expression through the inhibition of IFN-γ [41]. A previous study suggested that IL-4 and IL-13 can also induce sVCAM-1 levels [42] as IFN-γ can act as an antagonist in the induction of sVCAM-1 by IL-4 and IL-13 through competition for JAK2 trans-membrane receptors [43]; inhibition of IFN-γ was suspected to further increase sVCAM-1 levels.

Apolipoproteins such as Apo-A1 and Apo-E play an important role in lipid metabolism, particularly in cholesterol transport. A significantly reduced Apo-A1 level was found in the PCAD subjects. It is, thus, our expectation that Apo-A1 was the main component of HDL that was involved in the reverse cholesterol transport [44]. A similar association between Apo-A1 and the PCAD subjects was also noted by previous researchers [45,46]. Interestingly, the Apo-A1 level was found to be significantly higher in the OCAD subjects when compared to the same-aged controls and the PCAD subjects, presenting discordance with the supposed anti-atherogenic properties of Apo-A1. We believe that, in aging, some of the Apo-A1 may be functionally impaired; thus, an increase in the Apo-A1 level may not exert any protective effects. Indeed, studies have shown that the function and distribution of Apo-A1 in the plasma are markedly different from those found in the artery [47]. In addition, a study on Apo-A1 recovered from the human atheroma and plasma revealed a potent proinflammatory activity on endothelial cells [48]. Therefore, as aging correlates positively with oxidative stress, it could be possible that an increase in plasma Apo-A1 concentrations will not reflect their functionality within the arterial wall.

An elevated Apo-E level was observed in the OCAD subjects when compared to the age-matched control group. This could be a protective situation for OCAD patients as an in vivo study revealed that Apo-E-deficient mice exhibited higher levels of plasma cholesterol and subsequently developed atherosclerotic lesions [49]. Treatment with Apo-E mimetics successfully reduced cholesterol levels and protected mice against atherogenesis, suggesting anti-atherogenic properties for Apo-E [49]. However, an optimal level of Apo-E would be required for maintaining cholesterol levels. In fact, only a small amount of Apo-E (40 µg/dL) is needed to effectively lower plasma cholesterol [50], while overexpression of Apo-E3 in Apo-E-deficient mice was found to lead to hypertriglyceridemia [51].

Homocysteine and CRP are well known as CAD biomarkers. Homocysteine promotes atherosclerosis by reducing nitric oxide (NO) production, inducing inflammation and oxidative stress while promoting monocyte adhesion to the endothelium [52]. Homocysteine was suggested to be an independent risk factor for CAD, and the risk was seen to be higher in men [53,54]. However, there was no significant difference for homocysteine levels between both the PCAD and OCAD subjects and controls. On the other hand, an elevated level of homocysteine was seen in the OCAD subjects when compared to the PCAD subjects. One plausible explanation could be that homocysteine levels may be associated with the number of vessels affected [55]. Our OCAD subjects were more likely to have multiple vessel disease with a higher percentage for triple vessel disease as compared to PCAD (56% vs. 41%). Contradicting the findings with homocysteine, CRP levels were seen as higher in both cases when compared to their age-matched controls, thus justifying their selection as a CAD biomarker. In addition, we showed that CRP was a better biomarker exclusively for PCAD after adjusting for multiple variables. Other studies have also demonstrated a positive correlation between CRP and PCAD [56] and age [57].

An increase in vitamin D levels was found in PCAD compared to age-matched controls. Previous studies suggested reduced vitamin D as one of the risk factors for CAD, while our results appeared to contradict this notion [58,59]. However, it is important to note that vitamin D levels may be affected by environmental factors including diet and period exposed to sunlight. This could partly be the reason contributing to an increased vitamin D level in PCAD. Moreover, although reduced vitamin D is associated with CAD, excess vitamin D levels have been suggested to increase CRP concentrations [60]. Therefore, it is possible that inflammatory properties of vitamin D can only occur at certain optimum concentrations while reduced or excess vitamin D may contribute to disease progression.

## 5. Future Direction

CAD progression was affected by multiple components including genetics, diet, and lifestyle; thus, evaluating cases based on one biomarker might be inappropriate. Future research should venture into introducing a panel of biomarkers that may evaluate the risk and predisposition to PCAD. In addition, a well-designed prospective study should be conducted to establish a biomarker that can be used routinely. Currently, only CRP was used as one of the indicators for CAD in a clinical setting. Our study also highlighted the necessity to identify cases as PCAD or OCAD during clinical evaluation. It is of the utmost importance to properly select the right biomarker based on the age of the individuals in order to predict the risk to CAD. Nevertheless, our biochemical data for PCAD and OCAD may provide the foundation for studies to further justify their candidacy as potential biomarkers.

## 6. Limitation of the Study

The small sample size influenced the data significantly but, according to our sample size calculation, our sample size was significant for elucidating differences between PCAD and OCAD categories. Our healthy samples were also limited to those who were considered normal as determined by ECG. Those who went for an angiogram, although with normal results, were not considered as healthy controls since they presented with symptoms. In addition, it was difficult to recruit healthy, asymptomatic individuals from angiography procedures especially when involving older subjects. The study also included only Malay males as the subjects since it was important to increase the homogeneity of our subjects as race and gender may influence CAD progression significantly. Males were selected for this study as the male gender is a known risk factor for PCAD [23,61]. The Malay population was selected as there are limited studies in this population while studies involving Indian and Chinese subjects have been performed previously [62,63]. Some of the biochemical parameters, especially those that are involved in inflammation among CAD cases, can be possibly elevated during the pre-cardiac event time point and temporally resolved. However, our blood samples were taken prior to angiography or PCI procedure, thus limiting the beneficial effect from intervention that may resolve certain biomarkers.

## 7. Conclusions

In this study, we demonstrated a different biochemical profile seen with and potential biomarkers for PCAD and OCAD cases. A panel of elevated sVCAM-1, IL-6, and CRP and reduced Apo-A1 levels has the potential to be a biomarker for PCAD, while a panel of increases in IL-2, CRP, and Apo-E biomarkers is suitable for OCAD. Overall, an elevated CRP was found to increase the risk up to 2.47 times for PCAD, while an elevated IL-2 was shown to increase the risk up to 1.64 times for OCAD cases.

## Figures and Tables

**Table 1:** Demographic and biochemical profiles of subjects. Data are presented as mean ± SD. Categorical data are presented as n (%). ^a^ *p* < 0.05 compared to C45; ^b^ *p* < 0.05 compared to C60; * data were analyzed using a *t*-test; ** data were analyzed using a Mann–Whitney test.

	C45	PCAD	*p*-Value for PCAD vs. C45	C60	OCAD	*p*-Value for OCAD vs. C60	Normal Value
Demographic Data							
Age	37.96 ± 7.31	41.60 ± 5.98	*p* = 0.058 *	66.77 ± 6.05	64.87 ± 3.96	*p* = 0.156 *	-
Height (m)	1.68 ± 5.48	1.65 ± 6.17	*p* = 0.148 *	1.62 ± 4.26	1.62 ± 6.09	*p* = 0.760 *	-
Weight (kg)	75.67 ± 18.12	79.07 ± 12.46	*p* = 0.415 *	73.04 ± 14.35	71.46 ± 9.47	*p* = 0.631 *	-
BMI (kg/m^2^)	27.17 ± 5.27	28.47 ± 4.03	*p* = 0.318 *	27.85 ± 5.25	26.94 ± 2.94	*p* = 0.44 *	19–24
Systolic blood pressure (mmHg)	125.54 ± 17.40	129.14 ± 20.86	*p* = 0.493 *	138.96 ± 15.03	146.80 ± 22.02	*p* = 0.122 *	120
Diastolic blood pressure (mmHg)	74.84 ± 15.30	81.45 ± 13.28	*p* = 0.092 *	76.38 ± 11.34	77.47 ± 12.67	*p* = 0.739 *	80
Fasting glucose (mmol/L)	4.90 ± 0.57	5.92 ± 1.11	*p* < 0.001 ^a,^*	6.19 ± 2.10	7.72 ± 2.93	*p* = 0.006 ^b,^**	3.5–5.5
Liver Profile							
Albumin (g/L)	46.65 ± 2.62	45.00 ± 3.95	*p* = 0.135 *	43.48 ± 3.74	42.41 ± 4.09	*p* = 0.309 **	35–52
Total protein (g/L)	76.12 ± 3.77	74.45 ± 5.19	*p* = 0.254 *	73.83 ± 5.43	71.55 ± 4.76	*p* = 0.095 *	65–85
Total bilirubin (µmol/L)	9.94 ± 4.29	10.69 ± 9.63	*p* = 0.765 *	10.93 ± 3.06	9.00 ± 4.35	*p* = 0.056 *	<24
ALT (U/L)	28.88 ± 8.54	38.10 ± 21.42	*p* = 0.046 ^a,^*	25.34 ± 3.74	32.17 ± 2.19	*p* = 0.301 **	<41
ALP (U/L)	72.24 ± 19.29	84.68 ± 24.03	*p* = 0.078 *	73.48 ± 14.67	79.62 ± 31.53	*p* = 0.348 *	<129
Renal Profile							
Sodium (mmol/L)	140.55 ± 1.80	138.33 ± 3.23	*p* = 0.002 ^a,^*	141.76 ± 2.67	138.00 ± 3.08	*p* < 0.001 ^b,^*	135–152
Potassium (mmol/L)	4.10 ± 0.43	4.38 ± 0.42	*p* = 0.013 ^a,^*	3.91 ± 0.43	4.48 ± 0.65	*p* < 0.001 ^b,^*	3.6–5.4
Urea (mmol/L)	4.36 ± 1.26	4.70 ± 1.75	*p* = 0.404 *	4.67 ± 1.02	5.88 ± 2.20	*p* = 0.011 ^b,^*	<8.3
Creatinine (µmol/L)	86.55 ± 20.97	92.40 ± 20.32	*p* = 0.281 *	98.00 ± 15.39	113.18 ± 44.07	*p* = 0.094 *	<130
Lipid Profile							
Triglyceride (mmol/L)	1.13 ± 0.48	2.57 ± 1.82	*p* < 0.001 ^a,^*	2.10 ± 1.31	1.86 ± 0.94	*p* = 0.834 **	<2.3
HDL (mmol/L)	1.29 ± 0.34	0.94 ± 0.27	*p* < 0.001 ^a,^*	1.25 ± 0.29	1.10 ± 0.29	*p* = 0.066 *	>1
LDL (mmol/L)	3.11 ± 0.81	2.66 ± 1.25	*p* = 0.114 *	3.60 ± 1.16	2.44 ± 1.04	*p* < 0.001 ^b,^*	<3.9
Total cholesterol (mmol/L)	4.92 ± 0.96	4.89 ± 1.58	*p* = 0.919 *	5.61 ± 0.93	4.58 ± 1.86	*p* = 0.011 ^b,^*	<5.2
Smoking							
Smoking, n (%)	13 (43)	20 (67)		2 (7)	8( 27)		
Number of arteries affected							
1, n (%)		15 (50)			11 (37)		
2, n (%)		4 (13)			3 (10)		
3, n (%)		11 (37)			16 (53)		
Lipid-lowering drug							
Lovastatin, n (%)		7 (23)			8 (27)		
Rosuvastatin, n (%)		2 (7)			4 (13)		
Atorvastatin, n (%)		8 (27)			5 (17)		
Simvastatin, n (%)		10 (33)			12 (40)		

**Table 2:** Pearson correlation between parameters in PCAD. ** Correlation was significant at *p* < 0.01; * correlation was significant at *p* < 0.05.

		CRP	Vitamin D	Homo- cysteine	sVCAM-1	sICAM-1	IL-2	IL-6	IL-10	Apo-A1	Apo-E
CRP	r	1	0.091	−0.108	0.283	−0.104	−0.025	0.212	0.251	0.044	0.151
	Sig.		0.664	0.625	0.214	0.655	0.908	0.333	0.227	0.837	0.493
Vitamin D	r		1	−0.120	0.335	−0.271	−0.051	0.021	0.436 *	0.241	0.120
	Sig.			0.552	0.094	0.200	0.795	0.914	0.016	0.208	0.541
Homocysteine	r			1	0.167	−0.057	−0.142	0.477 *	−0.026	−0.078	0.217
	Sig.				0.437	0.808	0.498	0.016	0.897	0.706	0.298
sVCAM-1	r				1	−0.164	0.177	0.630 **	0.734 **	0.327	0.328
	Sig.					0.489	0.407	0.001	0.000	0.104	0.117
sICAM-1	r					1	0.316	0.137	0.065	−0.385	−0.208
	Sig.						0.142	0.542	0.763	0.069	0.341
IL-2	r						1	0.103	0.059	0.472 *	0.069
	Sig.							0.617	0.765	0.013	0.737
IL-6	r							1	0.436 *	−0.019	0.224
	Sig.								0.020	0.927	0.270
IL-10	r								1	0.079	−0.011
	Sig.									0.685	0.957
Apo-A1	r									1	0.583 **
	Sig.										0.001
Apo-E	r										1
	Sig.										

**Table 3:** Pearson correlation between parameters in OCAD. ** Correlation was significant at *p* < 0.01; * correlation was significant at *p* < 0.05.

		CRP	Vitamin D	Homo- cysteine	sVCAM-1	sICAM-1	IL-2	IL-6	IL-10	Apo-A1	Apo-E
CRP	r	1	−0.272	0.058	0.478 *	0.032	−0.147	−0.094	0.289	0.120	0.058
	Sig.		0.221	0.789	0.018	0.889	0.492	0.671	0.181	0.567	0.789
Vitamin D	r		1	0.249	−0.232	−0.220	0.086	−0.266	−0.328	−0.010	0.122
	Sig.			0.220	0.253	0.291	0.682	0.199	0.110	0.962	0.554
Homocysteine	r			1	−0.014	−0.113	−0.131	0.081	−0.122	0.239	0.584 **
	Sig.				0.943	0.583	0.515	0.687	0.544	0.212	0.001
sVCAM-1	r				1	−0.150	−0.224	0.256	0.562 **	−0.107	0.100
	Sig.					0.466	0.262	0.189	0.002	0.579	0.614
sICAM-1	r					1	−0.262	−0.004	−0.263	0.151	−0.112
	Sig.						0.206	0.985	0.194	0.451	0.586
IL-2	r						1	0.134	−0.078	0.127	−0.047
	Sig.							0.513	0.704	0.520	0.818
IL-6	r							1	0.334	−0.178	0.345
	Sig.								0.096	0.366	0.078
IL-10	r								1	0.020	−0.072
	Sig.									0.921	0.720
Apo-A1	r									1	0.078
	Sig.										0.686
Apo-E	r										1
	Sig.										

## Data Availability

All data generated or analyzed during this study are included in this published article.
